# Advanced manufacturing and processing in the time of COVID‐19

**DOI:** 10.1002/amp2.10061

**Published:** 2020-07-03

**Authors:** Matthew Realff, Jan Lerou, Michael Rinker

**Affiliations:** ^1^ Chemical and Biomolecular Engineering Georgia Institute of Technology College of Engineering Atlanta Georgia USA; ^2^ Jan Lerou Consulting, LLC Hilliard Ohio USA; ^3^ Pacific Northwest National Laboratory Energy and Environment Directorate Richland Washington USA

The COVID‐19 pandemic has exposed fragilities and reinforced the resiliency of different elements of our manufacturing and processing systems. Food processing, in particular meat packing, currently a very labor‐intensive operation, experienced significant disruption and the car industry implemented shutdowns which will ripple through supply chains in the coming months. It became clear that packaging supply chains are bottlenecks as producers tried to divert food stuffs from restaurants to consumer stores, and shifts in demand for paper products caused persistent consumer outages. The basic chemicals and fuels industry did not see significant disruption, but wild price fluctuations did occur as reduced demands and an unfortunately timed production increase caused available storage capacity to be strained. Recycling industries saw, and will continue to experience, both ends of the disruption as their consuming industries reduced demand and their providers stockpiled material or diverted it to trash. The beverage industry has significant inventory in public venues that has expired. Boutique level hand sanitizer production has resulted as these beverage production systems have been reconfigured and the rules surrounding the grade of alcohol relaxed.^[^ ^1^ ^]^

We are sure there are many of you who have worked long hours to innovate your manufacturing and processing systems to cope with the challenges of COVID‐19 and we would welcome articles and commentaries on how advanced manufacturing can play a role in our collective response. In this issue of JAMP we share a commentary on the rapid manufacturing of vaccines as our contribution to understanding how our community might respond to COVID‐19,^[^ ^2^ ^]^ and we would like to encourage you to consider other ways in which our manufacturing systems will change in response to the pandemic. However, much as there is a need to address the short‐term disruptions caused by a pandemic, and to understand how research can respond to create advanced manufacturing and processing systems with flexibility and resilience in the future, we also need to continue to push forward with efforts that provide new foundations for those features and we have curated articles that we hope fulfill that goal.

Process intensification remains a key approach to advanced manufacturing enabling more nimble and responsive systems. This issue provides two examples tackling a variety of processes: the epoxidation of vegetable oils and the manufacturing of butyl acrylate.

Rahim et al.^[^ ^3^ ^]^ show that by using a mesoscale oscillating baffled reactor the process can be converted from a batch operation to a continuous one and in the process they estimate that the new reactor is approximately 144 times smaller than the batch reactor operating at the same production rate.

The Simulated Moving Bed Reactor (SMBR) technology has been an example for process intensification for a couple of decades. Constantino et al.^[^ ^4^ ^]^ have combined this technology with membrane pervaporation creating a Simulated Moving Bed Membrane Reactor which reduces the number of operation sections for the manufacturing of butyl acrylate while reducing the eluent consumption and improving the productivity. A complete analysis of all process metrics is given in this paper.

The use of Artificial Intelligence (AI) in the chemical process industry is being used in new ways that are challenging the way this industry should consider the adoption and deployment of Industry 4.0 that has become ubiquitous in other business sectors. Colegrove^[^ ^5^ ^]^ discusses the importance of protecting data as critical intellectual capital and how many in the AI vendor community do not understand the value of data to the process industry. The commentary offers insight on the need for chemical engineers and data scientists to work closely together to solve technical and operational problems that require analysis of data that is broader than process data and where fundamental models break down. This commentary takes on additional importance for resiliency in light of the social and economic impact of COVID‐19.
